# Exploratory trials, confirmatory observations: A new reasoning model in the era of patient-centered medicine

**DOI:** 10.1186/1471-2288-11-57

**Published:** 2011-04-25

**Authors:** José A Sacristán

**Affiliations:** 1 Clinical Research Department, Lilly Spain Avenida de la Industria 30,28108 Alcobendas. Madrid. Spain

## Abstract

**Background:**

The prevailing view in therapeutic clinical research today is that observational studies are useful for generating new hypotheses and that controlled experiments (i.e., randomized clinical trials, RCTs) are the most appropriate method for assessing and confirming the efficacy of interventions.

**Discussion:**

The current trend towards patient-centered medicine calls for alternative ways of reasoning, and in particular for a shift towards hypothetico-deductive logic, in which theory is adjusted in light of individual facts. A new model of this kind should change our approach to drug research and development, and regulation. The assessment of new therapeutic agents would be viewed as a continuous process, and regulatory approval would no longer be regarded as the final step in the testing of a hypothesis, but rather, as the hypothesis-generating step.

The main role of RCTs in this patient-centered research paradigm would be to generate hypotheses, while observations would serve primarily to test their validity for different types of patients. Under hypothetico-deductive logic, RCTs are considered "exploratory" and observations, "confirmatory".

**Summary:**

In this era of tailored therapeutics, the answers to therapeutic questions cannot come exclusively from methods that rely on data aggregation, the analysis of similarities, controlled experiments, and a search for the best outcome for the average patient; they must also come from methods based on data disaggregation, analysis of subgroups and individuals, an integration of research and clinical practice, systematic observations, and a search for the best outcome for the individual patient. We must look not only to evidence-based medicine, but also to medicine-based evidence, in seeking the knowledge that we need.

## Background

The fact that randomized clinical trials (RCTs) lend strength to causal inference explains why they are regarded as the paradigm for evaluating the efficacy of therapeutic interventions and the cornerstone of clinical epidemiology and evidence-based medicine (EBM) [1]. Phase I-IV clinical trials are the pillars that sustain the regulatory systems for the approval of new drugs. RCTs of adequate size and duration are required to test an *a priori *hypothesis (i.e. the hypothesis that a new drug is superior to placebo or to the standard treatment).

The main objective of RCTs is to assess the average efficacy of a therapeutic intervention in a group of patients. In clinical practice, treatment effect estimates obtained from RTCs are the basis for deciding how to treat individual patients. However, RCTs were not developed for the purpose of determining individual treatment. As a result, some clinicians are becoming increasingly concerned that the evidence obtained from RCTs, which reflects the average results observed in the population, is being applied to guide clinical practice [2].

Modern medicine is faced with the challenge of placing patients - rather than diseases, molecules or statistics - back at the center of the clinical universe [3]. Patient-centered care and comparative effectiveness research are two of the most important movements to have arisen in the field of medicine in recent years [4,5]. Both seek to determine which options are most effective for which patients [6].

Advances in pharmacogenomics have fueled great expectations surrounding the potential development of individually-tailored therapies, in keeping with the adage "one size does not fit all" [7]. Genomic signatures can facilitate patient stratification (i.e., risk assessment), treatment response identification (i.e., surrogate markers), and/or differential diagnosis (i.e., identifying who is likely to respond to which drug) [8].

A patient-centered approach to treatment requires the development of research methods and regulatory changes adapted to the paradigm of personalized medicine. This new "patient-centered research" should not strive to predict what percentage of patients will respond to a given intervention, but rather, to determine which patients will respond and what is the most appropriate intervention in each case.

The view that prevails today in therapeutic clinical research is that observations are useful for generating new hypotheses and that controlled experiments (particularly RCTs) are the most appropriate method for assessing and confirming the efficacy of interventions. This view, however logical it may appear from a regulatory perspective concerned with "average patients" and with the language of populations, does not necessarily hold true in a patient-centered medicine (PCM) approach, which is based on the language of individuals.

## Discussion

One of the main drawbacks of traditional RCTs is the lack of external validity of their results. These studies are conducted by expert investigators on relatively homogeneous patient populations under rigid protocol-driven "experimental" conditions in which concomitant medications are avoided. Hence, results from phase III RCTs are not always applicable to the heterogeneous populations of patients seen by clinicians in everyday clinical practice [9].

To minimize the lack of generalizability of the results of explanatory RCTs [10], some have proposed conducting large pragmatic or practical RCTs with wider selection criteria and more heterogeneous patients [11,12]. Not surprisingly, this approach has led to a dramatic increase in the sample size required to detect small differences in drugs' effects that, although statistically significant, may have little practical benefit on patient outcomes. A few years ago, several prestigious trialists envisaged the future of therapeutic clinical research as consisting of large RCTs with heterogeneous patient samples capable of detecting small differences in the response to the interventions being compared [13,14]. Predictably, in an atmosphere highly influenced by EBM, large pragmatic RCTs have come to be seen as the "logical" starting point for generating evidence for comparative effectiveness research [15].

Paradoxically, large RCTs and "megatrials" (sometimes including more than 10,000 patients) [16] with heterogeneous samples have not solved the problem of representativeness [17]; indeed, they have aggravated it. Clinicians know that some patients respond to a particular drug in a given therapeutic group and not to others, even though meta-analyses and megatrials have shown no differences among patients [18]. A focus on large RCTs prevents us from seeing the trees for the forest. The following question comes to mind: "Are large samples needed because the differences in effectiveness among the interventions being compared are small, or are the differences detected small because heterogeneity has a diluting effect on important differences among subgroups?"

We must change our way of reasoning: *clinical research is most urgently challenged by the realization that the fundamental problem with RCTs is not the lack of generalizability of their results, but their lack of "individualization." *From the perspective of patient-centered medicine, neither traditional phase III RCTs nor "real-world" RCTs can be considered "confirmatory," as they cannot tell us which options are most effective *for which patients*. The EBM movement has engendered the notion that randomization, evidence and truth are equivalent concepts [19], but in medicine, the "final" word is almost always temporary and medical "truth" has an expiration date [20]. We must banish the idea that large sample size equates to a higher level of scientific evidence, and hence, of truth. In a patient-centered health care system, personalized clinical research methods must be developed and the RCT, the paradigm under the model in which "one size fits all," cannot be the only option. New approaches should not rely on an analysis of the similarities among patients, but rather, of the differences among them. If the road from the individual to the average patient calls for aggregating data, the return trip should consist of exactly the reverse, i.e. data disaggregation, *the analysis of subgroups and individuals*.

### Subgroup Analyses

Subgroup analyses are conducted to evaluate the effect of a particular treatment on a specific endpoint in subgroups of patients who share a baseline characteristic [21]. Such analyses assess the heterogeneity of treatment effects in different groups of patients. They are useful if specific subgroups differ widely in their risk of a poor outcome depending on treatment or non-treatment; if important pathophysiological differences between subgroups could influence the effect of treatment; if there is uncertainty about when to treat, or if a particular subgroup is undertreated in routine clinical practice [22].

The limitations and dangers of *a posteriori *multiple subgroup analyses have been widely reported in the literature [21]. To be helpful, "analyses must be predefined, carefully justified, and limited to a few clinically important questions, and post-hoc observations should be treated with skepticism irrespective of their statistical significance" [22].

But even if we overlook the biases inherent to *post hoc *analyses and the fact that such analyses are eminently exploratory, what sense can there be in aggregating data from thousands of patients only to disaggregate them again later? The assessment of "absolute" effectiveness through megatrials conducted with heterogeneous patient samples should give way to comparative effectiveness research in homogeneous patient subgroups formed *a priori*, based on better an understanding of the factors that determine differences in prognosis and response to treatment among different patient subtypes.

Some believe that "with the revolution of predictive power, the approach that we should try to amass many thousands of patients in 'simple' trials simply to balance the unknown biologic parameters through randomization is less and less appealing in a medical world where mechanisms and predictors of disease are becoming revealed" [7]. In this context, "targeted" clinical trials could dramatically reduce the number of patients one would need to study when the mechanisms of action of a drug are understood and accurate biomarkers for responsiveness are available [23]. Ideally, the results of these "small" trials would prompt regulatory approval for use in subgroups of patients in whom use of the drug would have demonstrated a favorable risk-benefit ratio.

### Confirmatory observations

Individual observations play a central role in "personalized clinical research." Bradford Hill, considered the father of modern RCTs, warned that blind faith in experimentation and the loss of credibility of clinical observations would lead to a loss of significant knowledge [24]. Case reports and case series may be the weakest level of evidence, but they often remain the "first line of evidence" [25,26]. Individual cases are most useful when they show us the unexpected and, in research, the unexpected can signal a new truth [27].

The terms "inductive" and "deductive" reasoning have been highly controversial in the specialized literature [28], so I have tried to avoid them in this paper, although some allusions are inevitable. Induction has been defined as the "inference of a generalized conclusion from particular instances" [29]. Inductive reasoning usually involves generalization. Many patients are similar, but all patients are different. Similarities between patients explain why inductive reasoning, under which generalizations are drawn from an accumulation of cases, has taken such strong hold in research. "Deduction" is defined as "inference in which the conclusion about particulars follows necessarily from general or universal premises" [29]. The philosopher Karl Popper was decisively influential in popularizing deductive reasoning in scientific research [28].

In an excellent reflection paper about the gold standard role played by RCTs, Cartwright states that "the claims of RCTs to be the gold standard rest on the fact that the ideal RCT is a deductive model: if the assumptions of the test are met, a positive result implies the appropriate causal conclusion." She goes on to say that "from positive results in an ideal RCT... we can deduce that the causal hypothesis is true" [30]. This is the basis of the "confirmatory" nature of RCTs, from which population-average results are obtained.

**Figure 1:**
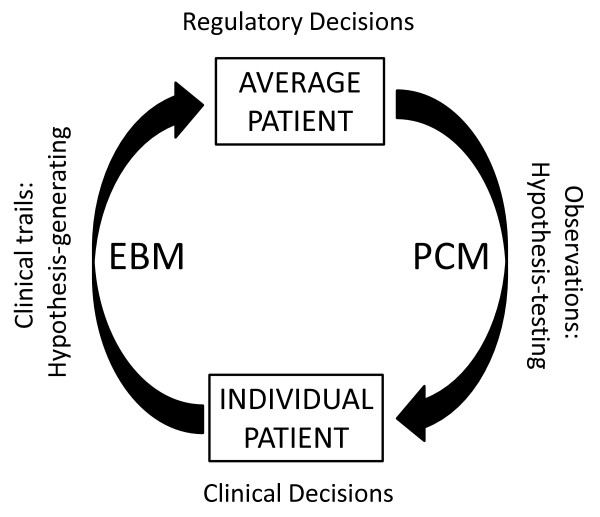
Directionality of scientific reasoning in evidence-based medicine (EBM) and patient-centered medicine (PCM)

The trend towards "individualization" calls for alternative ways of reasoning. No particular method should be regarded as a gold standard or as universally best [30]; the only gold standard should be, instead, whatever approach will yield the information the researcher needs [30], and the choice of clinical research method should fit the question for which an answer is sought [31,32]. A patient-centered research perspective demands a shift towards hypothetico-deductive logic, which holds up theory to the facts for verification and treats any hypothesis concerning the average patient as tentative until shown to be valid or not by the particular circumstances of individual patients or subgroup of patients. RTCs would thus be considered "exploratory" and observations "confirmatory" in nature [33]. In this patient-centered approach, RCTs would serve primarily to generate a hypothesis (i.e. that one intervention is superior to others in some types of patients), while observations would test the validity of the hypothesis for the individual patient (Figure 1). This directional shift in research rests on the tenet that asking better questions and developing the methods that will provide the answers, while routinely applying "destructive criticism" [34], are among the most productive ways to advance scientific knowledge [35].

The application of hypothetico-deductive logic should have an impact on drug R&D and drug regulation. Accordingly, the assessment of new therapeutic agents should be viewed as a continuous process, and regulatory approval should no longer be regarded as the final step in hypothesis-testing, but as the hypothesis-generating step. In reality, some of these changes are already taking place. More and more drugs are being approved subject to the subsequent provision of new effectiveness and safety data obtained under real life conditions, or to the development of risk management plans. What has been termed "real time regulation" [36] is a surefire indicator of the growing importance of the "hypothesis-testing" phase. Most current clinical therapeutic research is geared towards generating new hypotheses through RCTs. In the future, much greater emphasis should be placed on the testing of those hypotheses by means of real-life interventions.

It is widely known that in safety evaluation, individual cases can lead to the rejection of hypotheses. Many drugs whose risk-benefit ratio was initially rated as "acceptable" have been recalled following case reports of severe adverse events. However, the value of individual observations for testing hypotheses about efficacy is less obvious. Under the current model, based on RCTs and probabilities, the failure of a given patient to respond to a drug is not a surprising event. However, when dealing with tailored therapies, the failure of an entire series of patients to respond as expected (*i.e*., to not respond at all or respond to a higher dose or in a delayed manner) is an unusual and exceptional event and, as such, could prove extremely valuable in rejecting or modifying the hypothesis generated in the previous phase.

Under patient-centered research, patients and interventions should be assessed within the context of routine clinical practice. This demands that we attach greater weight to the "medical" component of research by integrating research and clinical practice and by realizing that all research and all clinical actions begin at the patient's bedside [37] and that every medical act is structured like an experiment [38].

Progressive implementation of electronic health records (EHRs) could greatly facilitate personalized clinical research. To date, EHRs have been used to carry out analytical observational studies of the average effectiveness of health interventions. Nonetheless, from a patient-centered perspective, they are potentially most useful not for effectiveness assessments requiring data aggregation, but rather, for disaggregating data and identifying differences among patients. The resulting information can be very valuable in responding to questions that differ from those typically formulated in RCTs.

EHRs can be used to (1) assess how and in which particular types of patients interventions are applied in clinical practice; (2) analyze different response patterns, identify patient subgroups and classify them in accordance with their risk factors and comorbidities; (3) help systematize the exceptions and the factors that condition their occurrence; or (4) support individual decisions by providing information about each patient [39]. Incorporating prediction rules, risk calculators, and decision aids in EHRs may facilitate decision-making at the individual level, with reliance on the benefits and risks anticipated for each patient [40]. If EHRs are to be employed routinely in PCM, data quality must be improved, formats must be standardized, and physicians must be encouraged to use them.

Systematized observations could also make it easier to implement some challenging and innovative ideas - one more instance of the potential applications of hypothetico-deductive reasoning. Some authors have suggested using prospective "formal case studies" to collect pure cases in whom to test *a priori *hypotheses [41]. In a planned case study, the investigator consciously and explicitly reflects on the theory and draws on it to develop a specific hypothesis or model that he subsequently tests in cases deliberately chosen to either confirm or reject it. Charlton et al. have used real-life examples to illustrate how formal case studies should be conducted [42].

*N *= 1 trials stand as another example of the potential "confirmatory" nature of individual observations, only in this case the design is experimental. Although analyzing such studies in detail is beyond the scope of this paper, some authors claim that they are among the purest forms of PCR and have been assigned first place in the evidence hierarchy [43]. These studies are the formalized equivalents of the "therapeutic trial" the physician so often conducts in the course of his everyday practice, with the huge advantage that patients benefit directly from the results of the research [44]. Although *n *= 1 trials are not feasible for all diseases, the potential of this type of design is probably not being fully exploited.

Finally, patient-centered medicine must be attentive to the patient's goals, preferences and values. In medicine, true individualization is grounded in respect for patients' preferences [45]. Hence, treatment choice should be tailored not only to clinical characteristics, but also to what the patient prefers. Psychological, social or cultural factors can alter disease prognosis in a given subject. Treatment adherence, level of tolerance for a particular adverse effect, past experiences, and health-related goals can all condition individual preferences and final health outcomes. Fortunately, one need not resort to sophisticated statistics or to the use of complex scales to find out what a patient prefers. Thoroughly having a thorough discussion with patients and asking them directly what their preferences are is an excellent strategy for conducting "personalized research".

## Summary

In the era of evidence-based medicine, RCTs have understandably been the paradigm of clinical research. In the era of patient-centered medicine, however, the notion of levels of evidence should be challenged to make room for a diversity of approaches. The shift towards tailored clinical research specifically demands that greater weight be given to the clinical components of the research process by integrating research and clinical practice and by drawing on the strengths of both observational and experimental methods. A patient-centered health care system is inconceivable without an individualized clinical research strategy. Regarding RCTs as exploratory and individual observations as confirmatory is one of the first steps that we can take in that direction.

We must not think of evidence-based medicine and patient-centered medicine as conflicting movements, but as complementary approaches. Modern medicine faces the fundamental challenge of reconciling the world of clinical guidelines, averages and risk-benefit for populations with the world of individual preferences and risk-benefit for individuals.

The choice of clinical research methods should be closely tied to the question for which an answer is sought. In the era of patient-centered medicine and comparative effectiveness research, the answers cannot come exclusively from methods based on the language of populations, such as RCTs; they must *also *come from methods grounded in the language of individuals [46] and driven by hypothetic-deductive logic, the value of observation, and a focus on optimizing individual patient outcomes. In short, knowledge must emerge not just from the world of evidence-based medicine, but also from the world of medicine-based evidence [47].

## Competing interests

I declare that I am employed by Eli Lilly & Company.

## Pre-publication history

The pre-publication history for this paper can be accessed here:

http://www.biomedcentral.com/1471-2288/11/57/prepub
